# Thiadiazines, *N*,*N*-Heterocycles of Biological Relevance

**DOI:** 10.3390/molecules17077612

**Published:** 2012-06-25

**Authors:** Hortensia Rodríguez, Margarita Suárez, Fernando Albericio

**Affiliations:** 1 Institute for Research in Biomedicine, Barcelona Science Park, Baldiri Reixac 10, Barcelona 08028, Spain; 2 Laboratory of Organic Synthesis, Department of Organic Chemistry, Faculty of Chemistry, University of Havana, Ciudad Habana 10400, Cuba; Email: msuarez@fq.uh.cu; 3 Department of Organic Chemistry, University of Barcelona, Martí i Franqués 1-11, Barcelona 08028, Spain; 4 School of Chemistry, University of KwaZulu-Natal, Durban 4041, South Africa

**Keywords:** heterocycles, thiadiazines, synthesis and biological activity

## Abstract

The 3,5-disubstituted tetrahydro-2*H*-1,3,5-thiadiazine-2-thione scaffold has found many applications in recent years. This review is aimed at highlighting the most important aspects of these compounds: Synthesis, spectroscopic characterization and biological activities. How the chemical nature of N-substituents influences the overall activity and cytotoxicity profile will also be discussed.

## 1. Introduction

**Figure 1:**
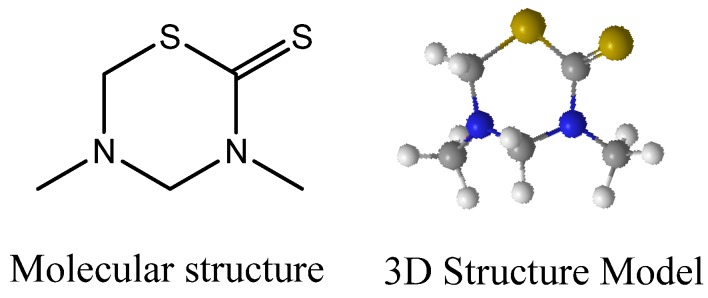
The 3,5-dimethyltetrahydro-2*H*-1,3,5-thiadiazine-2-thione scaffold.

Although the first representatives of the fully saturated 3,5-dimethyltetrahydro-2*H*-1,3,5-thiadiazine-2-thione scaffold (Figure 1) were synthesized for the first time in 1848 [1], their correct structure was not assigned until 1944 [2]. Until the 80s, some studies addressed the synthesis [3,4,5] and biological applications of these derivatives [6,7].

The antiprotozoal [8], antibacterial [9], antifungal [10], anthelmintic [11] and tuberculostatic properties [12,13] of tetrahydro-2*H*-1,3,5-thiadiazine-2-thione (THTT) have been known for several decades. In addition to its renowned antimicrobial activity, this versatile heterocycle has found an increasing number of applications in the drug research arena as a biolabile prodrug [14] in the design of drug delivery systems (DDSs) due to its high lipid solubility and enzymatic rate of hydrolysis. Moreover, the THTT scaffold has been used for arteriosclerosis treatment [15] and recently its application in antiepileptic pro-drugs has been reported [16]. In this regard, several amino acids [13,17], peptides [18,19], and primary-amine-containing drugs [20,21,22] have been successfully attached to the THTT moiety to enhance their cellular uptake by improving lipophilicity in the area where the drug molecule is released by the physiological and/or enzyme catalytic effects. Another important advantage of THTT derivatives is their stability in simulated gastric fluid (SGF), which facilitates their stomach absorption in a less ionized form than in the case of oral administration [17]. The excellent physico-chemical properties of this heterocycle have prompted its use as the main core in many integral projects for the development of new bioactive agents.

Due to the importance of this nucleus, the aim of this review is to highlight the synthesis and biological activity of the THTT scaffold reported in the last years, moreover the influence of the chemical nature of *N*-substituent on the overall activity and cytotoxicity profile. The main interest of these compounds is due to their higher versatile biological activity, which, at the same time, can be conjugated to other activities when THTT derivatives are used as DDS.

## 2. Synthesis of Tetrahydro-2*H*-1,3,5-thiadiazine-2-thione (THTT) Derivatives

**Figure 2:**
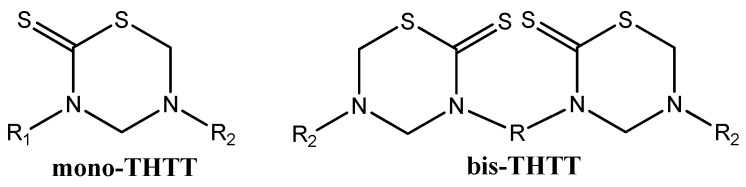
THTT structures.

In recent decades, the main studies on THTT derivatives have taken into account the molecular structure of these molecules to obtain an improved activity/cytotoxicity relationship [8]. In this context, we found many reports of the synthesis of several compounds with one THTT ring as the central core, with a great variety of substituents on the N-3 and N-5 position (mono-THTT) [8,9,10,13,19,21,22,23,24,25,26]. To enhance the biological effect, two THTT rings, connected to each other via their N-3 atom by a linear or branch aliphatic backbone and bearing alkyl or carboxyalkyl residues at N-5 (bis-THTT), have recently been incorporated into the same molecular structure (Figure 2) [8,27,28,29,30].

### 2.1. Synthesis of 3,5-Disubstituted-tetrahydro-2H-1,3,5-thiadiazine-2-thione (Mono-THTT)

**Scheme 1:**
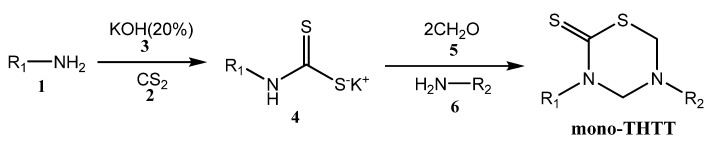
Synthesis of mono-THTT scaffold.

The most used procedure to obtain THTT derivatives in moderate or high yields is the reaction of the appropriate amine **1** with carbon disulfide (**2**) and potassium hydroxide (**3**) to give the dithiocarbamate potassium salt **4** (which was not isolated). This is followed by cyclocondensation with formaldehyde (**5**) and the selected amino acids [10,13,23,24,25], pseudo peptides [10,13,23,24,25,26], and amines or amino esters **6** [21] able to provide the nitrogen atom at N-5 of the thiadiazine ring. In the first step of these synthetic procedures, water was used as a protic polar solvent to stabilize **4**, while in the second step phosphate buffer at pH 7-8 was used (Scheme 1) [10,11,12,13,23,24,25,26].

This methodology allowed new synthesized mono-THTT derivatives to cluster in at least ten series. These derivatives differed in the nature (lipophilic or hydrophilic) of the substituent at N-3. Some series bore an aromatic (furfuryl [13,23], benzyl or benzyl derivatives [13,31] and D- or L-deacylated chloramphenicol [21]) and alkyl or cycloalkyl (ethyl, butyl, octyl, dodecyl, cyclopropyl and cyclohexyl) [10,11,12,13,23,24,25,26] moieties, all of them belonging to a lipophilic group; for others the starting amines were hydrophilic (carboxyalkyl) groups [23].

**Scheme 2:**
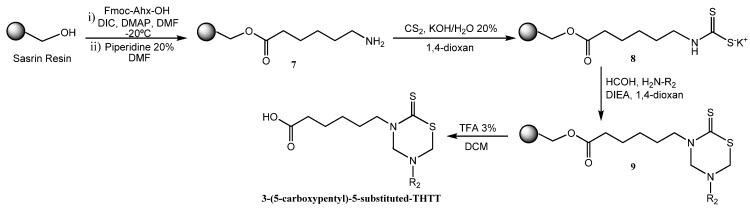
Solid phase synthesis of mono-THTT scaffolds.

Another synthetic method used to obtain these compounds was the solid-phase synthesis of 3-(5-carboxypentyl)-5-substituted tetrahydro-2*H*-1,3,5-thiadiazin-2-thione derivatives [32] (Scheme 2). *N*-Fmoc-protected 6-amino-*n*-hexanoic acid (Fmoc-Ahx-OH) was attached via its C-terminal to hydroxymethyl polystyrene using a ‘SASRIN’ linker. The bound amino acid **7** was transformed into the corresponding dithiocarbamate **8** followed by cyclization in the presence of formaldehyde and the corresponding free amino acids to afford 3-(5'-carboxypentyl)-5-substituted tetrahydro-2*H*-1,3,5-thiadiazin-2-thiones **9**. The final products were cleaved from the resin and obtained in moderate yields as a result of low solubility of the corresponding free amino acids in 1,4 dioxane (Scheme 2) [32].

However, the use of the solid phase methodology is limited by two factors: the possibility for the starting amines to be properly functionalized for efficient coupling to the resin and the low solubility of the amino acids in the solvent used. The development of this methodology would allow the generation of a combinatorial library for THTT compounds.

### 2.2. Synthesis of Alkyl-linked-bis-(2-thioxo-[1,3,5] thiadiazinan-3-yl) carboxylic Acids (Bis-THTT)

**Scheme 3:**
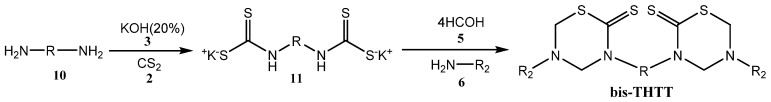
Synthesis of bis-THTTs scaffold.

Less information about the synthetic methods to obtain bis-THTTs [27,28,29,30,31,32,33,34,35] has appeared in the literature than for mono-THTTs [27,28,29,30]. The general procedure is very similar to that used for mono-THTTs. The bis-THTTs were obtained using diamines **10** and the amounts of all reagents were duplicated. In the first step the diamine **10** reacted with carbon disulfide (**2**) in the presence of potassium hydroxide (**3**) to obtain the expected bisdithiocarbamate salt **11**. The addition of formaldehyde (**5**) and the corresponding amine or amino acid **6** to **11** resulted in the cyclocondensation in a slightly alkaline medium (phosphate buffer, pH 7–8) to generate the desired bis-THTTs**,** after treatment with 15% HCl (Scheme 3) [27,28,29,30].

This procedure allowed the synthesis of new bis-THTT derivatives to cluster in at least four series, taking into account the starting diamine (1,6-diaminehexane, 2,2-dimethyl-1,3-propanediamine, ethane-1,2-diamine and ethyl 2,3-diaminopropanoate). All compounds were obtained in moderate to high yields, except when 2,2-dimethyl-1,3-propanediamine was used. This finding could be attributable to the use of a bulky diamine and the resulting steric hindrance at the cyclization stage.

**Scheme 4:**
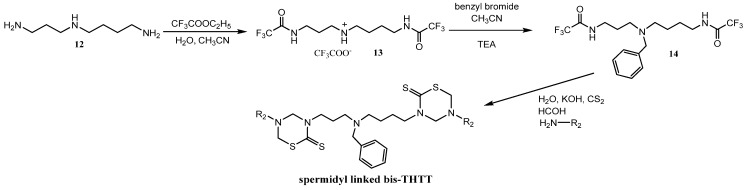
Synthesis of spermidyl linked bis-THTT scaffold.

The feasibility of synthesizing new bis-THTTs using more complex polyamines as linkages than the initially reported diamines was recently explored [30]. The N4-benzyl polyamine **14** was previously synthesized following a method reported by O’Sullivan *et al*. [33] via a protection-deprotection strategy using ethyl trifluoroacetate as the selective protective group for primary amines in the presence of secondary amines. The spermidyl-linked bis-THTT derivatives were obtained as solids in moderate yields. The synthetic route leading to spermidyl linked bis-THTT derivatives from the benzylated spermidine was similar to the one described above (Scheme 4).

### 2.3. Proposed Reaction Mechanism

**Scheme 5:**
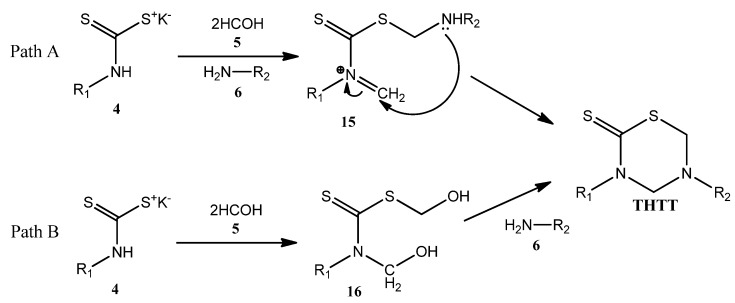
Proposed mechanisms for the THTT ring formation.

According to the authors [10,28], the formation of the thiadiazine ring is achieved via a one pot domino reaction between the pre-formed DTC, formaldehyde and the amino acid component. Despite being considered a multi-component reaction, the reactants are actually added in a stepwise fashion. Undoubtedly, one of the least explored aspects regarding the synthesis of THTT has been the reaction pathway from the corresponding DTC. In one approach (Scheme 5, Path A) the preformed DTC **4** is allowed to react simultaneously with formaldehyde and the corresponding amine to produce [substituted(aminomethyl)methanethionyl]methylidenazanium species **15** [28]. This species has two reactive centers in the same molecular backbone, a protonated imine and a thiosulfanylmethylamino group. The intramolecular addition of the secondary amino group to the carbon atom of the methylidene moiety leads to the THTT ring. The formation of this ring via a {[hydroxymethyl (substituted) carbamothioyl] sulfanyl}methanol intermediate **16** [10] was proposed (Scheme 5, Path B). This process involves in situ generation of **16** from the corresponding DTC **4** and formaldehyde (**5**), followed by condensation with a primary amine **6**. The isolation and characterization of an analog of **16**, via a crystallization process induced by the presence of KOH, was reported [10]. 

Recently, a preliminary DFT study aimed at predicting the probable cyclization mechanism of the thiadiazinane-2-thione from an intermediate of type **15** has been reported [34]. Based on experimental observations and DFT studies, a probable cyclization route to the THTT ring from the corresponding {[hydroxymethyl(substituted) carbamothioyl] sulfanyl}methanol intermediate **15** in aqueous medium has been proposed. Notably, water not only contributes to the reaction as a mere solvent, but also plays an active role in the reaction mechanism. 

### 2.4. Structural Characterization

Although numerous studies have been published on the synthesis and characterization of these compounds [12,23,35], it was only in 2001 that the first exhaustive structural characterization of mono-THTT derivatives was published [36,37,38]. These studies were considered an important structural data base to facilitate the characterization of novel compounds containing a THTT ring.

**Figure 3:**
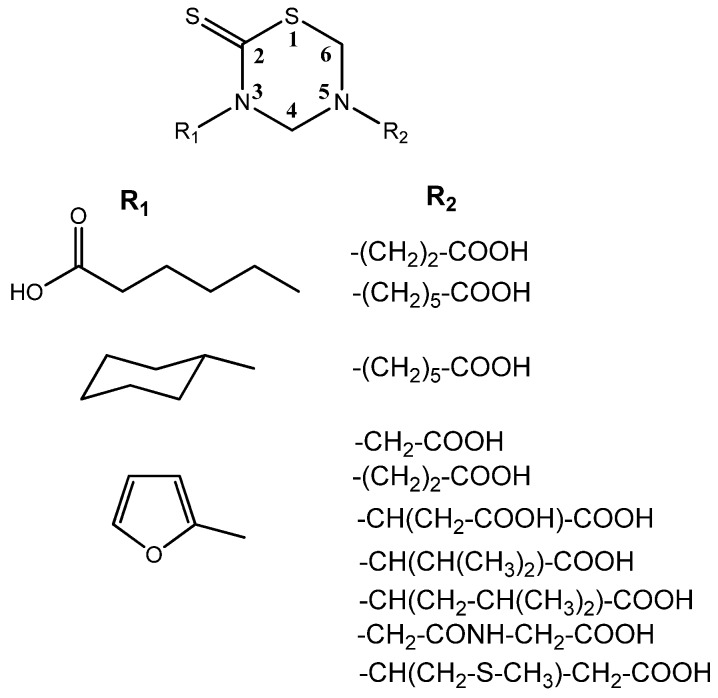
THTT derivatives characterized by NMR studies.

Nuclear Magnetic Resonance (NMR) studies deal with the complete ^1^H- and ^13^C-NMR assignments of a series of substituted THTTs (Figure 3) endowed with different organic addends on both heterocyclic nitrogen atoms. The 300 MHz ^1^H-NMR spectra of the THTT derivatives showed, in general, two singlets corresponding to the H-4 and H-6 ring protons around δ 4.50 and 4.40 respectively, in addition to other usual signals of the substituents. The ^13^C-NMR spectra of these compounds exhibited signals in the thiocarbonyl, carbonyl, aromatic and aliphatic regions. The thiocarbonyl carbon (C-2) in these systems appeared in the narrow range (δ 190.1–192.1 ppm), and the signals corresponding to the THTT ring was relatively insensitive to the nature of substituents on N-3 and N-5. In order to unequivocally assign all NMR signals, 1D and 2D techniques such as DEPT (135), HMQC and HMBC were used [36]. It is interesting that all systems showed a similar trend in the chemical shift of the common part of the molecular backbone for each type of compounds.

A structural study of 5-carboxy-ethyl-3-(2'-furfurylmethyl) tetrahydro-2*H*-1,3,5-thiadiazine-2-thione was made by means of X-ray crystallographic analysis. This study determined the most stable conformation in the solid state [37]. The theoretical calculations allowed chemists to gain a better picture of the conformational profile of the given compound by means of the semi-empirical AM1 method, as well as by ab-initio calculations at Hartree-Fock level using 3.21G* and 6-31G* basis sets. ^1^H-NOE experiments had also been carried out in order to obtain information about the conformational profile of this compound in solution [37].

Electrospray ionisation (ESI) in negative mode of pharmacologically significant mono-THTT derivatives, and their subsequent fragmentations using an ion-trap mass spectrometer were examined. Experiments on sequential product ion fragmentations (MS^n^) were performed in order to elucidate the degradation pathways for these compounds. The data reported show that the fragmentation of the even-electron [M−H]^−^ ions proceeds through an internal nucleophilic substitution displacement. Decarboxylation and extrusion of carbon disulfide were also observed [38].

Alternatively, the spectroscopic information gathered from previously synthesized mono-THTT derivatives [36,37,38] allowed confirmation of the structure of the bis-THTT compounds. The structures of all the bis-THTT derivatives reported in the bibliography were established on the basis of spectroscopic data [27,28,29,30,34,39]. In general the ^1^H- and ^13^C-NMR signals of each THTT-ring were undistinguishable and all series show a similar trend in the chemical shift of the common part of the molecular backbone. The ^1^H- and ^13^C-NMR spectroscopic data of alkyl, and polyamine-linked bis(2-thioxo-[1,3,5]thiadiazinan-3-yl) carboxylic acids, prepared from alkyl diamines and *N*4-(benzyl) spermidine, were fully assigned by the combination of one- and two-dimensional experiments (DEPT, HMBC, HMQC, COSY) [39].

## 3. Biological Activity of THTT Derivatives

Compounds derived from THTT have received particular attention due to their pharmacological properties. Numerous studies have been published on the antiparasitic properties of these derivatives [8]. Furthermore, these compounds also present antibacterial [9,13,22,25,26,27,28,31], antifungal [9,10,25,26,27,28,31], antiviral [7], and anticancer activity [24]. In addition, the high lipid solubility and ease of enzymatic hydrolysis [14] generally associated with this heterocycle have promoted its use as a biolabile prodrug in the design of drug delivery systems. The aforementioned properties and the possibility to attach several structurally distinct substituents to the heterocycle ring to modify either the biological or physico-chemical properties of these compounds prompted to use this heterocycle as a template in many research programs aimed at the development of new bioactive compounds. 

### 3.1. Antiparasitic Activity

The promising results of antiparasitic bioactivity of THTT derivatives could be attributed to the interaction of cysteine proteinases, present in most groups of parasitic protozoa [40], with isothiocyanates [41], generated by hydrolysis of the THTT ring in a protic medium [14]. Notwithstanding, the possible interaction of the released amino acids or dipeptides, attached to position 5 of the THTT ring, with other molecular targets, thereby enhancing the antiparasitic activity observed of these derivatives, should not be ruled out.

**Figure 4:**
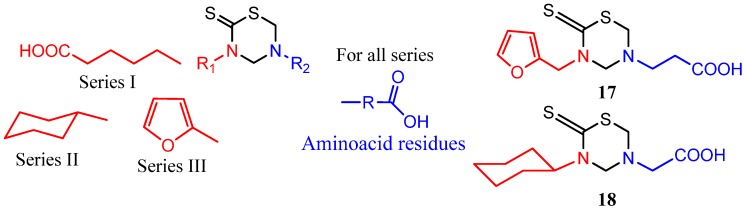
Mono-THTT derivatives tested against *T. cruzi* and *T. vaginalis*.

Some series of THTT derivatives have been studied as antiparasitic agents against *Trypanosoma cruzi*, *Trichomonas vaginalis*, *Leishmania amazonensis*, *L. donovani*, *T. brucei rhodesiens*, and *Plasmodium falciparum *[23,27,28,29,30,42,43,44,45]. Three series of mono-THTT were synthesized and tested against *T. cruzi* and *T. vaginalis *[23] (Figure 4). The series differ in the nature (lipophilic or hydrophilic) of the substituent at N-3 position and all derivatives showed significant *in vitro* antiprotozoan activity (both anti-trichomonas and anti-trypanosoma) at the highest dose tested (100 μg/mL). However, most of the compounds lost trichomonacidal activity at 10 μg/mL and only 5-carboxyethyl-3-(2'-furfurylmethyl) tetrahydro-2*H*-1,3,5-thiadiazine-2-thione (**17**) (Figure 4) maintained its efficacy at 1 μg/mL with anti-trichomonas activity similar to that of metronidazole. These results would indicate that the lipophilic character of R_1_ does not significantly influence the *in vitro* trichomonacidal activity. In contrast, compounds of series **II** and **III** showed trypanosomicidal activity, both at 100 and 10 μg/mL, whilst compound of series **I** only showed cytostatic activity at 10 μg/mL. The lipophilic substituents at N-3 showed better performance than hydrophilic ones for obtaining active compounds against *T. cruzi*, and at least six of these mono-THTT derivatives maintained trypanosomicidal activity at 1 μg/mL, showing a higher activity than nifurtimox (e.g., compounds **17** and **18**) [23] (Figure 4).

**Figure 5:**
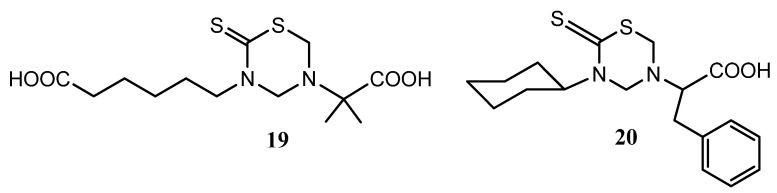
THTT derivatives with anti-amastigote activity.

Non-specific toxicity and anti-amastigote activity have been also reported for 24 mono-THTT derivatives corresponding to series **I**, **II** and **III**, and nifurtimox and benzidazole were used as reference drugs [42]. All the compounds were highly toxic at 100 μg/mL for macrophages and a few of them maintained this cytotoxicity even at 10 μg/mL. Of the derivatives assayed against amastigotes, 3-carboxypentyl-5-(α-dimethyl)carboxymethyl tetrahydro-2*H*-1,3,5-thiadiazine-2-thione (**19**) and 3-cyclohexyl-5-(α-phenyl)carboxymethyl tetrahydro-2*H*-1,3,5-thiadiazine-2-thione (**20**) (Figure 5) showed relevant activity, which was maintained at 1 μg/mL. Moreover, *in vivo* assays reported a reduction of parasitemia after the administration of **20** to infected mice [42].

Some mono-THTT derivatives of series **III** were tested *in vitro* for antiparasitic effects against both extracellular promastigotes and intracellular amastigotes of *L. amazonensis *[43,44]. The compounds were active against the amastigote form of the parasite, inhibiting parasite growth by 10 to 89% at a concentration of 100 μg/mL [43]. These results confirmed that the THTT compounds exert significant *in vitro* activity against *L. amazonensis *and indicated that some of them could be considered for further study as new therapeutic alternatives [43,44]. All the compounds evaluated caused an irreversible inhibition of promastigote growth either after 1h of treatment with 10 μg/mL or after 24 h with 1 μg/mL. However, the compounds exhibited high toxicity and inhibited phagocytosis in the murine host cell [44]. The mono-THTT compounds tested showed strong activity against *L. amazonensis* at low concentrations.

**Figure 6:**
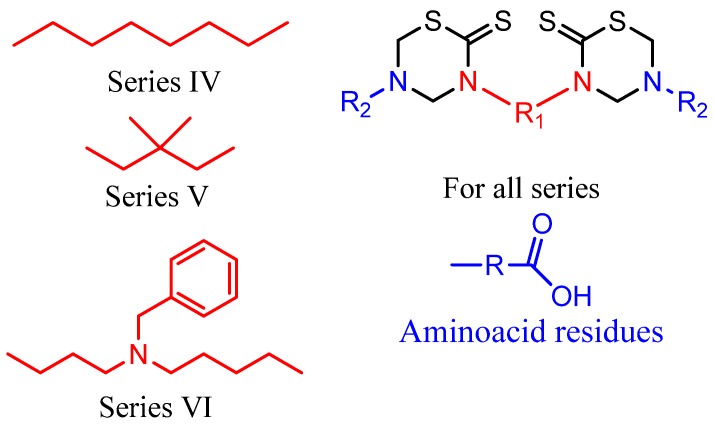
Bis**-**THTT derivatives with antiprotozoal activity.

To enhance the antiprotozoal effects, two rings were incorporated into the same molecular structure (bis-THTT). Three series of bis-THTT derivatives have been reported [27,28,29,30]. The *in vitro* activity of compounds belonging to series **IV** and **V** (Figure 6) against *L. donovani*, *T. b. rhodesiense*, and *P. falciparum* was studied. The best activity profiles were found against *T. b. rhodesiense*. It is interesting that the activity against the latter was enhanced for compounds with linear amino acid residues as substituents at position N-5 of the THTT ring. Despite exerting a notable activity against *T. b. rhodesiense*, derivatives belonging to series **IV** were more cytotoxic than the analogs of series **V** (Figure 6) [45].

The *in vitro* antiprotozoal evaluation of novel N4-(benzyl)spermidyl-linked bis-THTT derivatives from N4-(benzyl)spermidine (Series **VI**) was also disclosed [30]. These compounds showed a potent protozoocidal activity against *T. cruzi* and *L. donovani*, which in turns was comparable or greater than that of the currently used chemotherapies. Despite this observation, the novel structures displayed a higher cytotoxicity than previously synthesized alkyl ethers analogs with the same amino acidic residues attached to position N-5 of the heterocyclic ring. It has been hypothesized that increased cytotoxicity is related to interference with polyamine metabolism in mammals (Figure 6) [30].

To obtain THTT derivatives with potential antiparasitic activities, the lipophilic and hydrophilic nature of the substituents at N-3 and N-5 respectively, was important in order to improve the better structure-activity relationship.

### 3.2. Anticancer Activity

The highest cytotoxicity activity shown by some compounds of series I, II and III [23] may be indicative of potential anticancer properties. A selection of these compounds has been studied using cytotoxicity assays against HeLa, HT-29 and HepG2 cells, to evaluate their anticancer properties. The decomposition products of thiadiazinthione **16** have also been studied [24]. 

**Figure 7:**
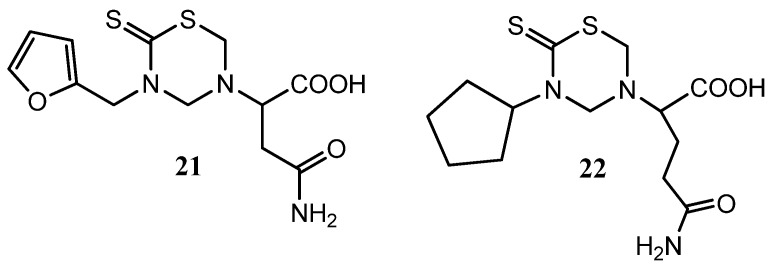
The promising THTT derivatives **21** and **22** for a future anticancer study.

Most of the mono-THTT derivatives showed noticeable cytotoxic properties against HeLa and HT-29 cells but not against HepG2 cells. The compounds of series I and II were, in general, less cytotoxic than those of series III, none of them showed an IC_50_ lower than 10 μmol against any cell line. The nature of R_1_ modulates the cytotoxicity of these compounds. Compounds bearing the aromatic furfuryl moiety (series III) yielded the most interesting thiadiazinones. However, the nature of R_2_ lesser degree influences in the cytotoxicity properties of these derivatives. The derivative 3-(2-furfuryl)-5-(α-carbamidomethyl)carboximethyl tetrahydro-2*H*-1,3,5-thiadiazine-2-thione (**21**) (Figure 7), bearing furfuryl and L-asparagine moieties, yielded the most interesting compound, which is a candidate for a future anticancer study [24]. These results allowed the application of QSAR methodology to study mono-THTT derivatives using the novel hybrid index pMRχ [46]. 

Recently, two series were synthesized to develop new cell cycle inhibitors [47]. Variable and promising *in vitro* antiproliferative activities were shown with the synthesized THTT derivatives. Compound **22** with with a 5-cyclopentyl group on N-3 and glutamine residue on N-5 of THTT moiety (Figure 7) showed the higher activity. There is no evident relationship between the cytotoxic activity of tested compounds and their lipophilicity.

### 3.3. Antibacterial and Antifungal Activity

Numerous studies have addressed the antibacterial and antifungal activity of THTT derivatives as prodrugs [9,10,13,22,25,26,27,28,31,48]. In general, it has been proposed that the antimicrobial activity of these compounds is based on isothiocyanates and dithiocarbamic acids, which are formed by the hydrolysis of the THTT ring [12].

**Figure 8:**
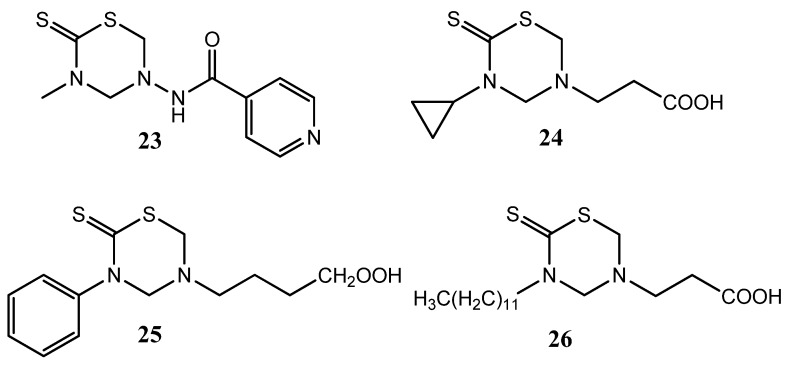
THTT derivatives with antuberculosis activity.

Isoniazid (INH) is still considered a first line drug for the chemotherapy of tuberculosis [22]. The THTT derivatives with INH attached at N-5 showed activity against *Mycobacterium tuberculosis*, but only the methyl derivative **23** was as active as INH (Figure 8). However, all these prodrugs showed greater antitubercular activity than INH when molar concentrations of the tested doses were considered [22]. Other THTT derivatives have been also tested as antitubercular agents and the compounds **24**, **25** and **26** showed the best performance. Moreover, the *in vivo* activity of compound **23** was also demonstrated (Figure 8) [13].

**Figure 9:**
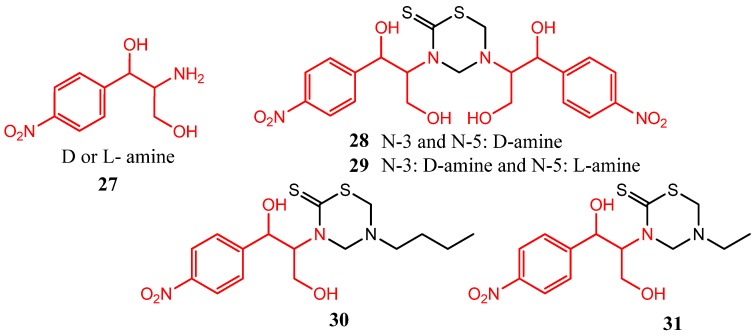
THTT derivatives with antibacterial and antifungal activity.

Antibacterial activity (against *Staphylococcus aureus*, *Escherichia coli*, and *Pseudomonas aeruginosa versus *chloramphenicol as reference) was achieved by introducing the deacylated chloramphenicol amine (D or L-amine) **27** at either N3 or N5 of the THTT system, as found for compounds **28**, **29**, **30**, and **31** (Figure 9) [9]. Furthermore, the presence of simple alkyl groups of these THTT systems at N-3 afforded moderate antifungal activity (against *Candida albicans*, *Trichophyton rubrun*, *Penicillium chrysogenum*, *Aspergillus flavus*, *Trichothecium roseum*, and *Drechslera halodes versus *Trosyd^®^ as reference) [9].

**Figure 10:**
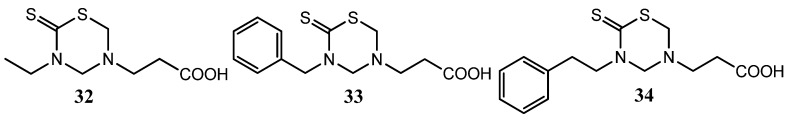
THTT derivatives of β-alanine with antifungal activity (against *C. albicans* and *F. oxysporum*).

Compound **32**, with a β-alanine residue at N-5 and ethyl group at the N-3 position of the THTT moiety, showed significant *in vitro* antibacterial activity (against *Bacillus cereus* and *Serratia rhadnii*) [25]. The β-alanine derivatives **33** and **34** bearing an aralkyl group at the N-3 position of THTT exhibited antifungal activity (against *C. albicans* and *F. oxysporum*) (Figure 10) [25].

**Figure 11:**
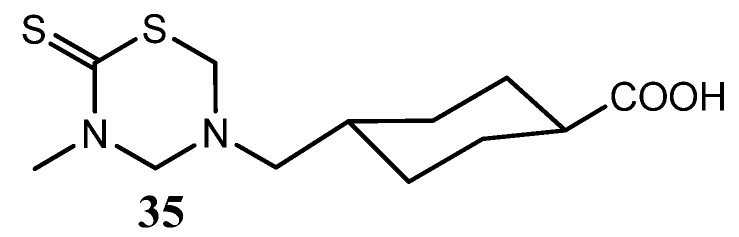
THTT derivative with antimicrobial activity against *S. aureus*, *B. subtilis*, *E. coli* and *P. aeruginosa*.

Ozcelik *et al*., synthesized a series of 3-substituted-5-(4-carboxycyclohexylmethyl)-THTTs that showed variable potencies against *S. aureus*, *B. subtilis*, *E. coli* and *P. aeruginosa* [26]. All these THTT derivatives exhibited potent antifungal activities against *C. albicans* and *C. tropicalis*. Among the synthesized compounds, **35** was the most effective compound with antimicrobial activity (Figure 11) [25].

**Figure 12:**
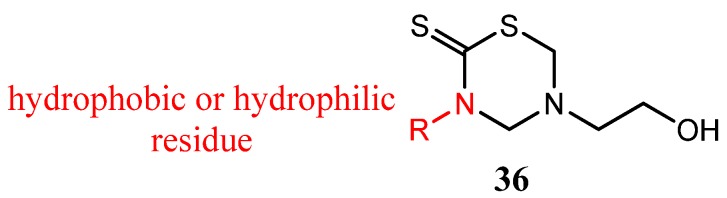
3-Substituted-5-(2-hydroxyethyl)-THTT derivatives.

Thirteen derivatives of 3-substituted-5-(2-hydroxyethyl)-THTT (**36**) were tested for their *in vitro* antibacterial and antifungal activity against some Gram positive and Gram negative bacteria and dermatophytic, saprophytic, phytopathogenic, and antagonistic fungi, respectively [48]. The results of bioactivity revealed the requirement of the lipophilic group at position N-3 and a polar substituent at N- for satisfactory antimicrobial activity (Figure 12) [48]. 

**Figure 13:**
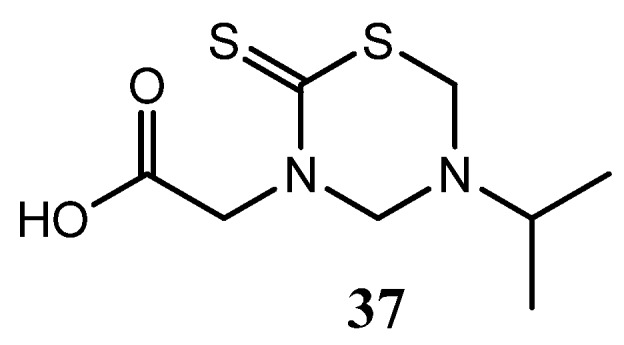
THTT derivative of glycine with antifungal activity.

Several THTT derivatives incorporating glycine and glycinamide were tested for their antifungal activity *In vitro* against *T. rubrum*, *C. albicans*, *P. expansum*, *T. hazianum*, *F. oxysporum*, and *A. flavus* [10]. The antifungal activity of these synthesized derivatives was greatly affected by the position carrying a polar group. The derivatives bearing this group at N3 were active, and the highest activity was observed with less bulky groups. Compound **37** showed the highest activity against the sporulation of most of the species tested (Figure 13) [25].

**Figure 14:**
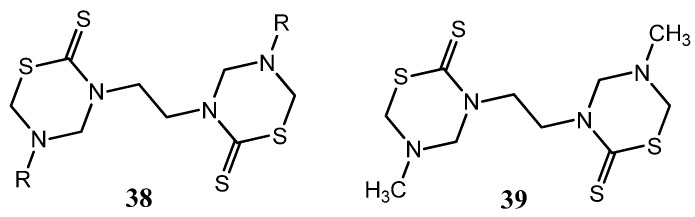
3,3'-ethylenebis(5-alkyl)-THTTs (**37**) tested as antifungal compounds.

In the search for promising antifungal compounds, nine 3,3'-ethylenebis(5-alkyl)-THTTs **38** were tested for their antifungal activity *in vitro* against *T. rubrum*, *C. albicans*, *P. expansum*, *T. hazianum*, *F. oxysporum*, and *A. flavus *[27]. The antifungal activity of these derivatives is greatly affected by the bulkiness of the side chain. The highest activity was obtained for compound **39**, which has the least bulky groups (Figure 14) [27].

**Figure 15:**
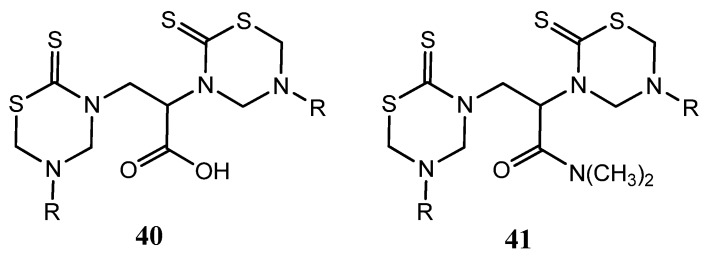
2,3-*bis*(5-Alkyl-2-thiono-1,3,5-thiadiazin-3-yl)propionic acids **40** and their corresponding *N,N*-dimethylpropionamides **41** tested as antibacterial and antifungal compounds.

Others two series of bis-THTT derivatives, 2,3-*bis*(5-alkyl-2-thiono-1,3,5-thiadiazin-3-yl)propionic acids **40** and their corresponding *N,N*-dimethylpropionamides **41 **(Figure 15) were screened *in vitro *against certain strains of Gram-positive and Gram-negative bacteria and compared with nalidixic acid and ciprofloxacin [28]. Moreover, the title compounds were tested for their antifungal activity *in vitro *against *C. albicans*, *P. expansum *and *T. hazianum*, and *A. flavus*. These compounds exhibited varied activity against the tested pathogenic bacteria and remarkable inhibitory effects on growth or sporulation of some of the tested fungal species [28].

**Figure 16:**
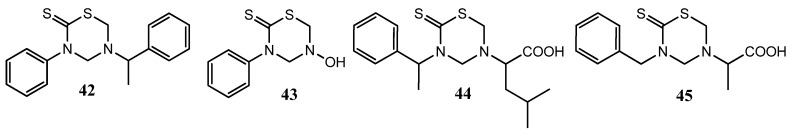
3-Phenyl-5-(substituted)-THTT derivatives **42** and **43**, and others THTT derivatives (**44** and **45**) tested as antibacterial and antifungal compounds.

Recently, a series of new THTT derivatives were evaluated for their *in vitro* antibacterial and antifungal activities by using the microdilution method in comparison with ampicillin and fluconazole [31]. 3-Phenyl-5-(1-phenylethyl)-THTT (**42)** and 3-phenyl-5-hydroxy-THTT **(43**) were found to be active against *Staphylococcus aureus* and *Enterococcus faecalis*, respectively. The antifungal activity of 3-phenyl-5-(1-phenylethyl)-THTT (**42**) against *C. krusei* and *C. parapsilosis* appeared greater than that of fluconazole, and this compound also exhibited antifungal activity against *C. albicans*. The antifungal activity of 3-(1-phenylethyl-5-[α-(isobutyl)carboxymethyl]-tetrahydro-2*H*-1,3,5-thiadiazine-2-thione (**44**) and 3-benzyl-5-carboxyethyltetrahydro-2*H*-1,3,5-thiadiazine-2-thione (**45**) against *C. krusei* were found to be similar to that of fluconazole (Figure 16) [31].

## 4. Conclusions

The most widely reported method to obtain THTT derivatives proceeds via a dithiocarbamate salt intermediate. This experimental procedure is simple and allows for a wealth of molecular diversity, depending on the nature of groups attached to the two nitrogen atoms of the heterocycle. 

The results presented in this review make it possible to analyze how the chemical nature of N-3 and/or N-5-substituents in the THTT ring potentially influences the overall activity/cytotoxicity profile against some microorganisms. Generally, lipophilic groups at both the N-3 and N-5 positions lead to compounds with high antimicrobial activity, but also high toxicity. The presence of a hydrophobic group at N-5 favored the antimicrobial activity of the THTT derivatives. In some cases, the introduction of two THTT rings in the same molecule strengthened their bioactivity. 3,5-disubstituted tetrahydro-2*H*-1,3,5-thiadizin-2-thione derivatives (THTTs) are of great interest for their biological and pharmacological activities, especially for their potential antiparasitic, antibacterial and antifungal properties.
